# Application of remote online learning in oral histopathology teaching in China

**DOI:** 10.4317/medoral.24441

**Published:** 2021-06-20

**Authors:** Yi Zhong, Wen Sun, Lili Zhou, Miaoning Tang, Wei Zhang, Jiani Xu, Yue Jiang, Laikui Liu, Yan Xu

**Affiliations:** 1 Jiangsu Key Laboratory of Oral Diseases, Nanjing Medical University, Nanjing, China; 2 Department of Basic Science of Stomatology, Affiliated Hospital of Stomatology, Nanjing Medical University, Nanjing, China; 3 Department of General Dentistry, Affiliated Hospital of Stomatology, Nanjing Medical University, Nanjing, China; 4 Department of Endodontics, Affiliated Hospital of Stomatology, Nanjing Medical University, Nanjing, China; 5 Department of Periodontics, Affiliated Hospital of Stomatology, Nanjing Medical University, Nanjing, China

## Abstract

**Background:**

The aim of this study was to investigate the application of remote learning and virtual microscopy in oral histopathology teaching, a unique experience in China. The oral histopathology teaching in Nanjing Medical University has been extraordinary.

**Material and Methods:**

98 third-year dental students of Grade 2016 took oral histopathology theoretical course face-to-face in 2019 (Traditional group). The 94 participants of Grade 2017 took online oral histopathology course using digital methods(E-Learning platform and Virtual Simulation Experiment Teaching Center for Dentistry) in 2020. During the practical laboratory sessions, the students in both Traditional group and Online group observed the same glass slides for morphological learning. A questionnaire survey explored students' attitudes towards the remote online learning. Results: The mean Theory test scores of the Online group (80.93±12.15) were significantly higher than those of the Traditional group (73.65±8.46) (*P* < 0.01). The mean total scores of the Online group (82.94±10.76) were significantly higher than those of the Traditional group (77.25±7.55) (*P* < 0.01). The percentage of high total test score (test score > 85) of the Online group (54%) was also significantly higher than that of the Traditional group (15%) (*P*< 0.01). Furthermore, both remote learning and virtual microscopy courses were well accepted by students according to the questionnaire.

**Conclusions:**

This study found that remote learning and virtual technology have a positive impact on oral histopathology. The findings reveal that the application of remote online learning has enhanced oral histopathology teaching in China.

** Key words:**Oral histopathology, dental undergraduate students, virtual microscopy, remote online learning, questionnaire.

## Introduction

Oral histopathology is a interdisciplinary discipline providing understanding of the nature and cause of diseases. It is one of the most important dental disciplines, connecting morphological changes to clinical work. Therefore, the study of oral histopathology is important in dental study (1).

It is true that COVID-19 pandemic limited the teaching all over the world in 2020. Higher education faced great changes owing to social distancing limitations (2-7). Due to the COVID-19 pandemic, the transition to remote learning tools is urgent (4-5). This has led to an immediate change to remote learning by a variety of universities. The oral histopathology teaching of Nanjing Medical University in China also faced unique challenges and took some actions. This study aimed to identify the approaches taken in Nanjing Medical University to deliver oral histopathology teaching.

To make the transition to remote learning easier, the oral histopathology teaching of Nanjing Medical University has applied the browser-based E-Learning and the virtual educational system for dental students accessible from off-campus that will help educators and students when they navigate remote learning. Students could choose to determine what access to the remote learning, such as computer, Tablet or mobile device. Indeed, the good online E-Learning platform of Nanjing Medical University has allowed students to finish after-class assignments, quiz and case analysis. Additionally, undergraduate dental students used the Virtual Simulation Experiment Teaching Center for Dentistry (VSETCD) of Nanjing Medical University for online learning at home. VSETCD is a good carrier of information and the open sharing of teaching resources undoubtedly. With the development of virtual reality technology, conventional stained slices are scanned and transferred into virtual records. Virtual microscopic slides have opened a new digital model of oral histopathology remote learning (8,9). The fast move to remote learning could keep students engaged in study solving the problem of teaching delay caused by social distancing limitation. Remote online learning represented an applicable educational methodology in oral pathology teaching.

It is convenient for universities to deliver online courses. In our study, all the theoretical lessons were video recorded and given through the Internet from February17th to April 18th for the new semester. Faced with the current situation with COVID-19, we have really kept students engaged in oral histopathology online learning. This paper discusses the application of remote learning and virtual microscopy in oral histopathology teaching in China.

## Material and Methods

- Participants and Setting

**Figure 1:**
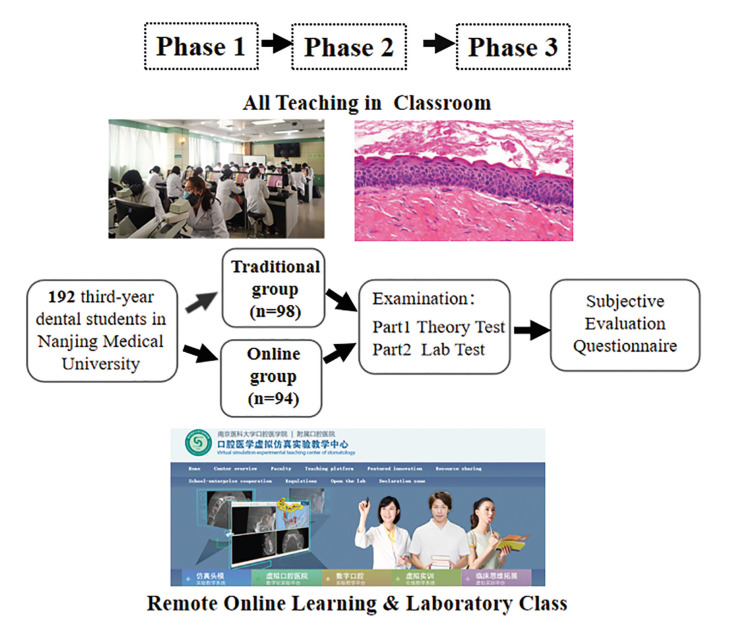
Flow chart of the practical process using remote learning for dentistry in this study.

In China, the 5-year dental undergraduate education programme is comprised of 4 years of preclinical training (year 1-4, including theoretical courses and practical courses) and 1 year of clinical training (year 4-5). This study was conducted at the School of Stomatology of Nanjing Medical University, China. The course comprised 22 theoretical lessons and 14 practical classes of histopathology distributed including both general and oral pathology. Oral histopathology was taught in the third and fourth academic year. 192 undergraduate dental students of Grade Three with the basic knowledge of histology and embryology were invited to participate in oral histopathology course. There were no differences in previous academic records between Traditional group and Online group. Fig. 1 showed the flow chart of the practical process using remote online learning for oral histopathology in this study.

**Figure 2:**
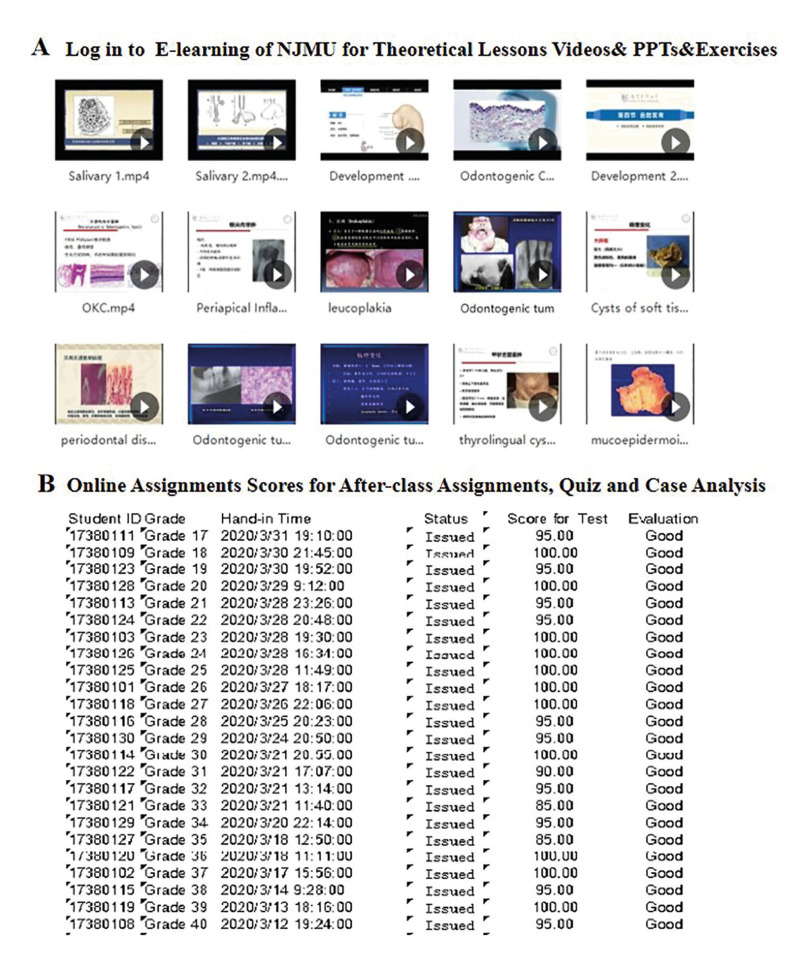
The application of E-Learning of NJMU for oral histopathology learning. A- Students can log in to E-Learning of NJMU for learning resources. B- Online assignments scores for after-class assignments, quiz and case analysis on the E-Learning of NJMU are presented.

**Figure 3:**
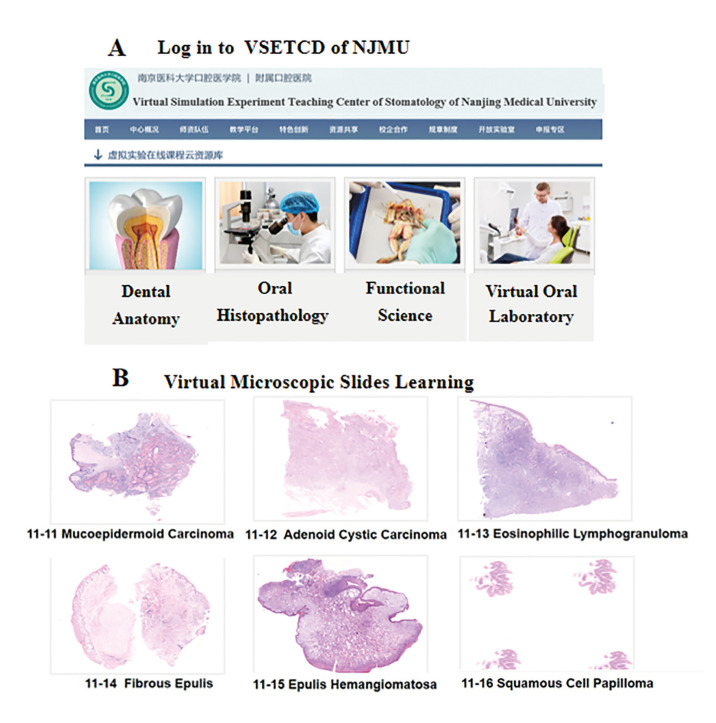
The application of VSETCD of Nanjing medical university for virtual microscopic slides learning. A - Participants did online oral histopathology learning using virtual simulation experiment teaching center for dentistry. B- Participants can observe microscope images on the computer, decreasing and increasing magnification by zooming in and out only with mouse.

For phase 1, in our study, 98 third-year dental students of Grade 2016 took oral histopathology course face-to-face in 2019 (Traditional group). The rest 94 participants of Grade 2017 (Online group) took online oral histopathology theoretical course using digital methods (E-Learning platform and Virtual Simulation Experiment Teaching Center for Dentistry) in February and March of 2020. For theoretical courses, dental undergraduate students of the Online group could download theoretical courses through the E-Learning platform of Nanjing Medical University (Fig. 2). Students must log in to the E-Learning platform to further finish a learning task list for each class. We have incorporated case analysis exercises to help students gain a clinico-pathological point of view (Fig. 2). In addition, participants in Online group logged in and did virtual learning freely with virtual microscopic slides supported by Virtual Simulation Experiment Teaching Center for Dentistry of Nanjing Medical University (Fig. 3). The students could use Internet Explorer for logging in and assessing the virtual slides. Each student was able to visualize the digital slides independently using the VSETCD (Fig. 3). As this is a new and emerging field of our oral histopathology curriculum, a member of the teaching team was online to welcome students to the online-learning room and make sure everyone can have lessons successfully and ensure each student still get interactivity like face-to-face teaching.

For phase 2, in April, when COVID-19 pandemic was under control in China, all the students of the Online group in Nanjing Medical University all went back to the classrooms for traditional laboratory study as Traditional group students did, a shift back to traditional optical microscope methods. In the face-to-face laboratory class students were required to use optical microscope to observe characteristics of the stained slides and draw pictures by themselves. During the practical laboratory sessions, the students in both Traditional group and Online group observed the same glass slides for morphological learning.

**Figure 4:**
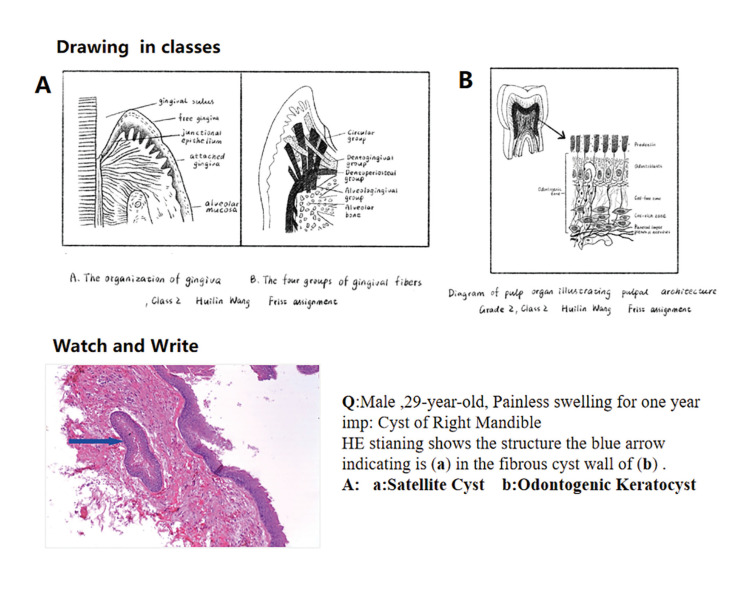
Evaluation of the drawings and lab course of oral histopathology. A,B- Representative students' drawings of histopathological structures. C - Representative temple of watch and write test.

For phase 3, students took tests including Theory Test and Lab Test (Fig. 4). Finally, all the students of the Online group filled out the questionnaires in the study at the end of the semester. The participants provided informed written consent, and the study followed the Declaration of Helsinki and the guidelines of the Ethics Review Committee of Affiliated Hospital of Stomatology of Nanjing Medical University with regard to medical protocols and ethics.

- Utilization of VSETCD

Virtual Simulation Experiment Teaching Center for Dentistry was applied for morphological learning for dental undergraduate students of the Online group (Fig. 3). The images were scanned using NanoZoomer Digital Pathology. The Digital Teaching Section of Oral Histopathology was organized by the Oral Pathology Committee of Chinese Stomatological Association and applied to the oral histopathology teaching through VSETCD. In this digital teaching section system, students can observe microscope images on the computer by zooming in and out with mouse to decrease and increase magnification (Fig. 3).

- Assessment Quizzes

Post-tests were composed of a set of Lab test and Theory test. The Lab test included the evaluation of students' drawing of histopathological structures and structure identification (Fig. 4). In each lab class, students drew typical histopathological structures on experimental report. In the Lab test, the Watch and Write tests were mainly presented in the form of PPT, combined with clinical manifestations (Fig. 4). Typical microscopic images were given through the computer and students answered as required. The theory test for the final exam included single-choice questions (30 items, 1 points each), identification sections (5 items, 4 points each) and essay questions (5 items, 5 points each), which mainly covered basic knowledge of oral histopathology (Fig. 1). Test questions were designed by a non-investigator from a third party based on the teaching syllabus of Oral Histopathology published by People's Medical Publishing House in 7th edition. Total scores for each student included regular score (20%) and final exam (80%). Regular scores included three parts: the drawing scores [5 '], online assignments scores for after-class [5 '] and the test scores of the Lab test [10 '] (Fig. 3, Fig. 4). A combination of course assessment and final assessment is emphasized throughout the course. During the period of marking, identity information of any participant was sealed to avoid bias, and each score point was double-checked by two examiners. Examiners were instructed and calibrated on the evaluation process prior to initiation. Files containing private information were deleted immediately after data collection.

- Questionnaire

The study data were collected in June 2020. A questionnaire was conducted with the students after completion of the study, which were used to demonstrate their responses and feedbacks towards the remote online learning experience of oral histopathology learning. The sum of the questionnaire points per subject was obtained. The questionnaire included the enjoyment and learning efficiency about remote online learning.

Responses from digital group were sorted and analyzed by two independent researchers (1=strongly disagree, 2=somewhat disagree, 3= neither agree nor disagree, 4=somewhat agree, and 5=strongly agree).

- Statistical Analysis

Data was analyzed using the statistical software program SPSS 22.0 for Windows software (SPSS, Inc., Chicago, IL, USA). Differences of the test scores among the different groups were evaluated by two-way ANOVA and independent-sample T tests. Statistical significance was defined as *P* < 0.05. The procedure was approved by the Ethical Committee Board of School and Hospital of Stomatology, Nanjing Medical University.

## Results

- Combination of E-Learning and VSETCD Enhances Oral Histopathology

A total of 192 dental students were included in the study, and none of the students were missing at different stages of the study. The sample was composed of 192 dental students attending the third academic year of the undergraduate dental programme in 2020 (Online group, n = 94) and 2019 (Traditional group, n = 98). The survey revealed students were aged from 20 to 23 years (average age of 22) in the study. The total score was 100 for each student. The score for daily performance was 10 for each student, which included the evaluation of students' drawing performance and on-line course, making up 10 per cent of the total score. The total score for Lab test was 10 for each student, which evaluated students' grasping of structures, making up 10 per cent of the total score. In the laboratory course of oral histopathology, students need to draw the microscopic manifestations of typical section and deepen their understandings of typical structures through drawing. The paper score for Theory test was 100, which mainly covered basic knowledge of oral histopathology, making up 80 per cent of the total score.

In the laboratory course of oral histopathology, students in the Online group exactly raised more questions compared to the students in the Traditional group. Indeed they valued the opportunity to solve the problems through face-to-face communication. As shown in Table 1, the test paper score, the score for on-line courses and for Lab test shall be added to the total score. The mean test paper scores of the Online group (80.93±12.15) were significantly higher than those of the Traditional group (73.65±8.46) (*P* < 0.01). The mean total scores of the Online group (82.94±10.76) were significantly higher than those of the Traditional group (77.25±7.55) (*P* < 0.01). The percentage of high total test score (test score > 85) of the Online group (54%) was also significantly higher than that of the Traditional group (15%) (*P*< 0.01). There were no differences of the Lab test scores between the Online group (8.13±1.75) and the Traditional group (8.28±6.48)(P> 0.05). There were no differences of the total scores between the male students (80.54±9.90) and the female students (83.71±10.87) in the Online group (*P*> 0.05).

- Students’ Attitudes towards the Remote Online Learning

Participants were asked to rate their perceptions of the learning experience with remote online learning at the end of their study. The surveys were tailored for the 94 third-year dental students in Online group. As shown in Table 2, responses were sorted and analyzed. A comprehensive breakdown of all questions and the constitutional ratios of responses were shown in Table 2. Nearly 85.1% (n=80) of the students strongly agreed that they could gain knowledge well via E-Learning and VSETCD. Only one (1.06%) partially disagreed with this statement. 87 (92.26%) out of 94 students strongly agreed that watching the online video lessons and virtual microscopy made it easier for students to review and understand the essentials of the lessons compared with the conventional method. Furthermore, both E-Learning courses and VSETCD were well accepted by students.

**Table 1:**
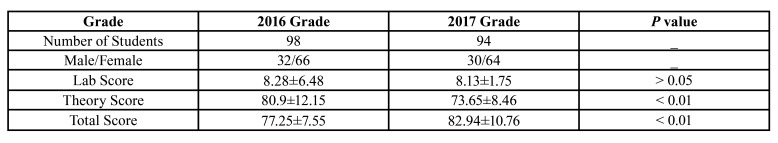
The assessment outcome of students in our study.

**Table 2:**
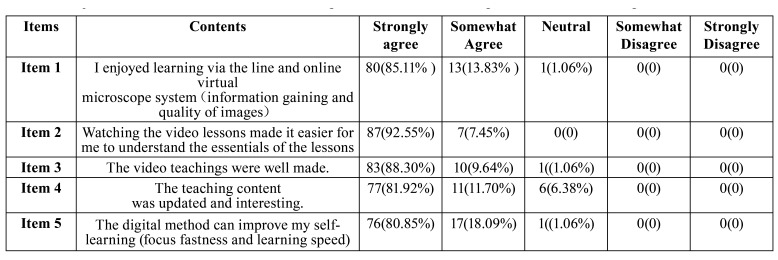
The questionnaire items for the students concerning their attitudes towards using the remote online learning.

## Discussion

In recent years, with the development of science and technology, the integration of various resources in the teaching has become the development trend (2). Augmented and virtual reality technology will represent a promising tool for dental education in the future (10). With the interruption of COVID-19 pandemic, dental institutions are confronted with great challenges in teaching. As the COVID-19 pandemic limited the face-to-face teaching in the classroom in February 2020, we delivered the theoretic lessons remotely online rather than hold face-to-face sessions with students in the classroom. In our study, online-based oral histopathology education has gained gradual acceptance among undergraduate dental students.

The E-Learning platform can provide a stage for sharing and discussion E-Learning has become an important method in dental education, providing education with lower cost at any time and anywhere (1,11-13). It has also been found to satisfy different teaching and learning styles. Most higher education institutions are moving to implement online teaching in their educational programs (12,13). Students of the Online group could download theoretical lessons through the E-Learning platform of Nanjing Medical University. Students could also log in to the E-Learning platform to finish exercises and case analysis for each class for the training of clinical thinking.

With the development of virtual reality technology, conventional stained slices are scanned and transferred into virtual records. Over the years, some pathology teaching departments have been integrating modern teaching methods including problem-based learning and computer-based methods (such as virtual microscopy and online cases) into their curricula (9,14,15). The digital pathology has also been validated for both diagnosis practice and pathology teaching (16,17). Also, oral histopathology has developed from an autopsy and macroscopy based discipline into a technically histological discipline applied in the teaching field (18). The microscopic digital interactive system, which integrates microscopic image, text, animation and flexible interaction, has been applied to oral histopathology virtual learning these years (1,19,20). The Virtual Simulation Experiment Teaching Center for Dentistry of Nanjing Medical University has been focusing on the construction of dental information-based experimental teaching resources and projects. Oral histopathology is just one part of the virtual simulation experiment system. We ensured a very collaborative network environment for learning to upgrade teaching quality. It is noteworthy that with the digital creative interaction system, students could share typical and interesting images synchronously. On the VSETCD platform, students could consult and discuss the histological findings of the images carefully. Moreover, students always contacted with the instructor for each class on VSETCD.

Compared with the traditional offline teaching, online teaching has its unique focus and concerns in the oral histopathology teaching. The prompt convenience of the online teaching and the high quality of the virtual microscopic slides offered an opportunity to identify microscopic structures for better learning. The remote teaching model could meet the needs of students freely without the limitation of space and time. Our students could take advantage of the unique opportunity to learn online theoretical lessons and review various typical virtual microscopic images in their own spare time, which is essential to the good grasp of knowledge. According to the report of the students, the application of virtual learning has enabled students to repeatedly view virtual images, which could help students consolidate key knowledge points and reinforce their learning after classes. The transition to remote learning has kept students engaged in study solving the problem of teaching delay caused by social distancing limitation. In general, dental undergraduate students have benefited from this online teaching mode of teacher-student interaction and image sharing, consistent with the literature (19).

In the present study, 94 dental undergraduate students took part in our newly designed oral histopathology remote learning. All the teachers and students cherished the precious opportunity for remote learning. Consequently, a higher interaction with the students in the classes was observed by the teacher. On one hand, the academic evaluations showed the test scores of the Online group were significantly higher than those of the Traditional group. On the other hand, a questionnaire to evaluate the students’ overall satisfaction with integrated teaching in oral histopathology was applied. According to the questionnaire of the experimental group, high level of interest and acceptance was documented among undergraduate dental students in the study. The questionnaire results showed 85.1% of the students strongly agreed that using remote online learning with better reviews of the images were better for study than traditional method. Overall, the responses of remote learning and virtual microscopy in the Online group were positive. These findings are in agreement with many other studies that have reported a positive impact of E-Learning on dental education (21).

To sum up, improved student performances were observed when online learning and virtual microscopy courses were introduced into oral histopathology teaching in Nanjing Medical University. The response of the current oral histopathology teaching to the COVID-19 crisis has been extraordinary. The results in the present study demonstrated that the application of remote learning and virtual microscopy has enhanced oral histopathology teaching in China. We could imagine that, social distance limitation will no doubt be in place in the near future until a COVID-19 vaccine is developed. This in turn has posed an impact on all the activities carried out in dental education. Currently, this continuous crisis will lead to many new collaborations of teaching methods. Remote learning, the new method, has been vital to help cope with the social distance limitation caused by this current crisis. There is still great potential for its further development and improvement.
